# The development of the cure of the functional intestinal disorder based on the differences of gut microbiota in aged patients: A randomized clinical trial

**DOI:** 10.1097/MD.0000000000027696

**Published:** 2021-11-05

**Authors:** Toshihiro Matsuura, Kazuhiro Kyokane, Satoru Yamada, Yuji Kuno

**Affiliations:** Department of Gastroenterology, National Center for Geriatrics and Gerontology, 7-430 Morioka-cho, Obu, Aichi, Japan.

**Keywords:** chronic constipation, gastrointestinal symptoms, intestinal immunity, laxatives, probiotics

## Abstract

**Background:**

Constipation, which is not an organic disease in the lower gastrointestinal tract, is a gastrointestinal symptom characteristic of elderly patients. Complaints of dyschezia increase with age, and it is difficult to treat in many cases. This study aimed to determine the appropriate treatment and its effects on intestinal immunity in elderly patients experiencing chronic constipation.

**Methods:**

Patients experiencing difficulty defecating were randomly divided into 2 groups. Group A was given only laxatives, whereas Group B was given laxatives combined with probiotics as an intervention. Both groups were compared based on the degree of improvement in constipation and its effects on the intestinal environment.

**Results:**

There was a significant improvement in constipation of elderly patients when probiotics were administered in combination with a laxative, suggesting that it may be a more effective treatment. Furthermore, the changes in the intestinal flora, examined before and after the intervention, tended to be associated with improvement of constipation.

**Conclusion:**

The results indicated that the improvement of intestinal flora was somewhat achieved by relieving constipation. Because intestinal bacteria significantly influence intestinal immunity and, thus, systemic immunity of the entire body, the development of better treatments for constipation would help to improve both the intestinal environment and immune function in the elderly.

## 1 Introduction

It has been reported that intestinal flora, which varies across individuals, is established at approximately 3 years of age. As the elderly age, it gradually changes due to environmental factors such as eating habits.^[1,2]^ Research has verified that harmful bacteria, such as *Clostridium perfringens*, increase with age, whereas useful bacteria, such as lactic acid bacteria, decrease.^[1]^ In conjunction with this transition, the older the patient, the more they suffer from constipation,^[3]^ suggesting that changes in the intestinal flora may be associated with abnormal bowel movements. Although individual differences exist in the composition of intestinal bacteria with age,^[4]^ it has recently been considered that these bacteria might affect intestinal mucosa and peristaltic movements, causing abnormal bowel movements.^[5,6]^ Many types of drugs can improve stool abnormalities; however, in many cases, their use is determined by the treatment policy/criteria of individual doctors, which often differs in each facility. However, the treatment of constipation is often refractory despite the administration of these drugs. In addition, laxatives, such as magnesium oxide preparations, may cause neurological symptoms associated with hypermagnesemia in the elderly with potential renal dysfunction; thus, caution is recommended before use.^[7]^ Conventionally, probiotics are prescribed to replace toxic bacteria and regulate the intestines to resolve ailments that cause diarrhea, such as bacterial enteritis. However, they have also been considered useful in the treatment of constipation. Moreover, it has been reported that probiotics facilitate ideal improvement of intestinal flora and can be used as a remedy for elderly people with an increasing number of harmful intestinal bacteria that are assumed to cause constipation.^[8,9]^

Therefore, we aimed to determine the appropriate treatment and its effects on intestinal immunity in elderly patients complaining of constipation. Patients experiencing difficulty with defecation were randomly divided into 2 groups. Group A was given only laxatives, and Group B was given laxatives combined with probiotics as an intervention. Both groups were compared based on the degree of improvement in constipation and its effects on the intestinal environment.

## 2 Methods

Participants included chronic constipation patients who were 65 years or older and were treated at the Department of Gastroenterology, National Center for Geriatrics and Gerontology. Informed consent, including consent for publication, was obtained from the participants before the study. According to International diagnostic criteria by Rome IV,^[10]^ chronic constipation was defined from the perspective of constipation symptoms and their persistence. Organic diseases in patients’ large intestines were eliminated by colon examination, which was necessary for determining the cause. For the treatment of abnormal stools, patients were assigned with block randomization by numbered container method to either the group given only laxatives (Group A) or the group given laxatives combined with probiotics (Group B). Personnel unrelated to the study managed the container and concealed the sequence until interventions were assigned, although the attending physician assigned the group with the subject's consent. Group A was administered the usual laxative medication Lubiproston, which acts as a chloride channel activator for the intestinal epithelium of the small intestine (the stimulant laxatives Sennoside and Sodium Picosulfate Hydrate can be used as a single dose). Alternatively, Group B was administered Lubiproston with a probiotic (Lactomin preparation) (the stimulant laxatives Sennoside and Sodium Picosulfate Hydrate can also be used as desired). The degree of improvement in stool was compared and examined between Group A and Group B. By analogy with the preliminary results of 5 trial cases, registration of a total of 60 cases, 30 cases each in both groups, was considered appropriate. These intervention trials began in August 2017 following approval by our hospital's Ethics and Conflicts of Interest Review Committee (No1066), and registration was completed in March 2020 as originally planned.

Basic patient information, such as age, sex, duration of illness, medical history (especially with or without neurological disease and diabetes), lifestyle habits, and dietary habits, were collected. Nutritional status was evaluated using a mini nutritional assessment. Because patient activities of daily living (ADL) are expected to affect stool abnormalities, basic ADL was evaluated using the Barthel Index as a life function survey. Furthermore, the mental status of patients was assessed using the hospital anxiety and depression scale (HADS) due to a potential relationship between the psychological state of the patient (such as depression) and constipation.^[10]^

In addition, we tested patients’ blood (2 bottles of 10 ml) to assess their nutritional status and collected their stool to establish the distribution of intestinal bacteria. Intestinal bacteria were outsourced for metagenomic analysis using a next-generation sequencer using 16S rDNA detected in stool as an index (TechnoSuruga Laboratory Co., Ltd).

As the primary endpoint, the degree of improvement in spontaneous defecation was investigated for 12 weeks from the intervention time. Comparison over time was examined using paired *t* test, and comparison between the 2 groups was verified using Cox test because of the small number of cases. In addition, patients self-reported their defecation status by recording the number of defecations per week, the Bristol score as stool properties, the degree of improvement in subjective symptoms, and the properties of the stools. Furthermore, changes in the intestinal flora before and after the intervention were also examined in both groups.

## 3 Results

The entries were completed by the end of September 2019. Group A included 13 cases, although 3 dropped out from the initial 16 cases, and Group B included 17 cases with no dropouts. Since the drug used was approved for insurance coverage, no adverse events occurred in the participating subjects. Group B comprised more women and patients with a long average history of constipation. There were 2 patients with a fairly long history of constipation in group B, 360 months and 600 months, so there was a large difference in the mean values. However, no significant difference was observed between basic patient information, including nutritional status, ADL, and age distribution (Table 1).

**Table 1:** Patient background.

	Group A	Group B
Age	77.8 ± 6.1	76.0 ± 4.3
Sex	Male 6; female 7	Male 6; female 11
History of constipation	68.5 ± 71.5 mo	108.2 ± 169.0 mo
MNA score	25.0 ± 1.8	24.1 ± 2.86
ADL	97.0 ± 8.3 points	97.8 ± 5.1 points

All items except gender are displayed as average values ± STD. ADL = activities of daily living, MNA = mini nutritional assessment.

**Figure 1:**
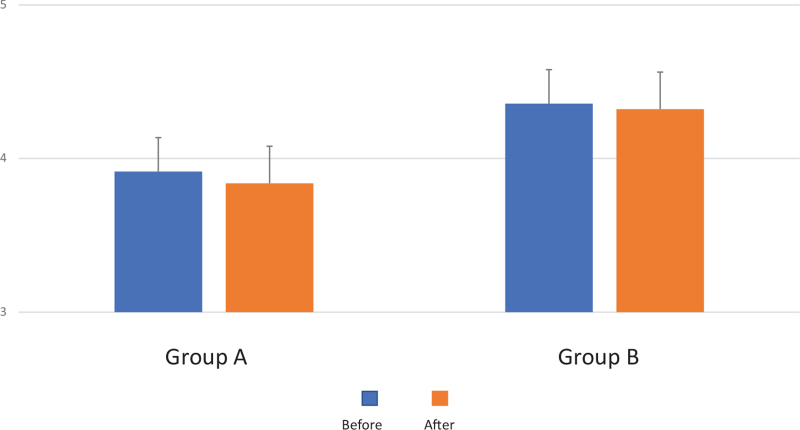
No clear difference was observed in Group A or Group B, although nutritional status was examined for albumin levels before and after drug intervention.

Nutritional status was screened using mini nutritional assessment; however, no patients with obvious malnutrition were identified in the cases examined in this study. In addition, both groups’ nutritional status before and after drug intervention was examined for albumin levels; however, no clear difference was observed in either group (Fig. 1).

**Figure 2:**
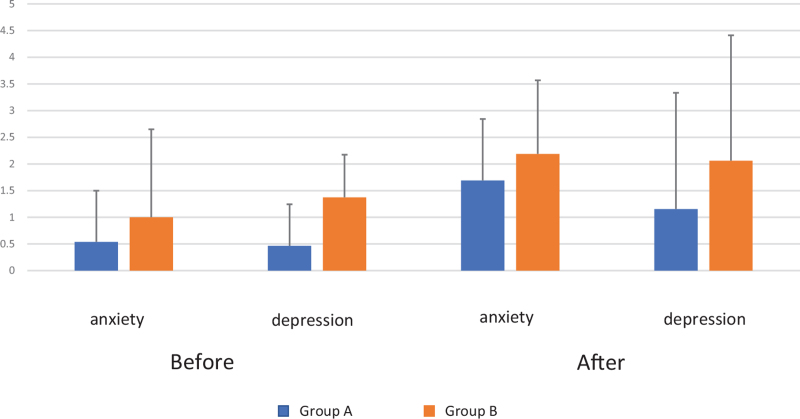
No change in the scores before and after the intervention was identified, although patients’ quality of life was examined using the HADS score. HADS = hospital anxiety and depression scale.

Patients’ psychological status was examined using the HADS score. The anxiety and depression scores of most patients in both groups were as low as 0 to 2. However, Group B included 1 case with a depression score of 9 points. There was no change in the scores before and after the intervention (Fig. 2). The HADS score was expected to improve by relieving constipation; however, it could have been affected by the results of the colon examination, which revealed that there were no organic diseases in the intestinal tract. Patients’ life function survey was evaluated by assessing the basic ADL using the Barthel Index, and both groups were evaluated as good conditions with similarly high scores (Table 1).

**Figure 3:**
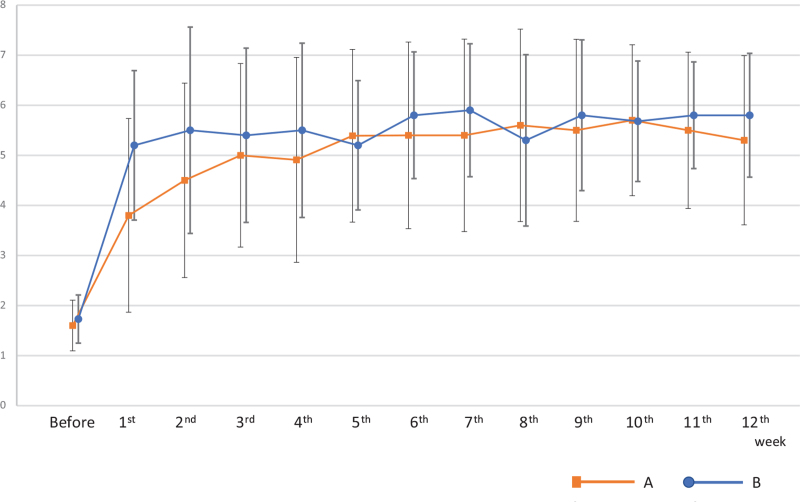
The number of defecations, which were nearly the same after the third week, significantly increased in Group B compared to Group A in the first week after the intervention (*P* < .05). Moreover, a significant (*P* < .01) improvement was observed in both groups after the intervention in the final 12th week compared to the first week.

Next, we examined the degree of improvement in spontaneous defecation, which is the primary endpoint. Before the intervention, the number of defecations per week for both groups was similar: 1.6 times per week in Group A and 1.7 times per week in Group B (Fig. 3). However, the number of spontaneous defecations in Group A increased to 3.8 times and that in Group B to 5.2 times during the first week after the intervention. Thus, the number of defecations significantly increased in Group B compared to Group A (*P* < .05, Cox test) during this week. Although there was no significant difference in the second week, the number of spontaneous defecations in Group B still tended to be high. Moreover, no significant changes were obtained at all points after the third week until the 12^th^ week. During the final 12^th^ week, the number of defecations per week in Group A was 5.3 times, and that in Group B was 5.8 times. These results reveal a significant (*P* < .01, paired *t* test) improvement in both groups after the intervention.

**Figure 4:**
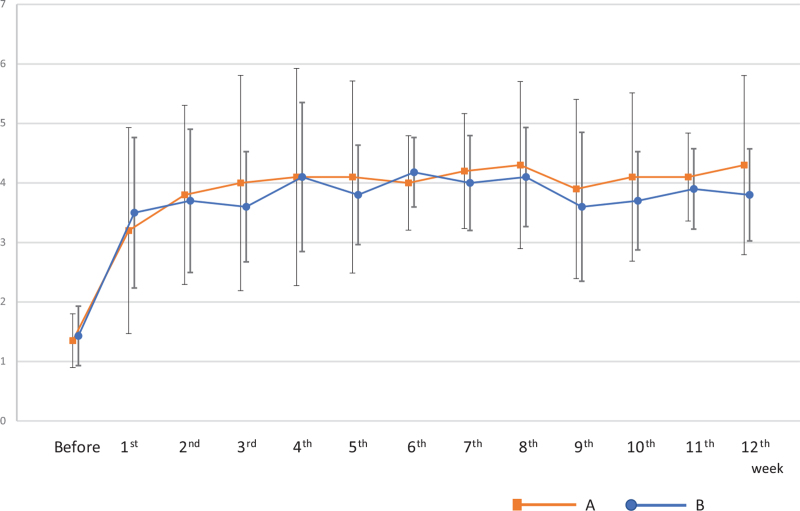
There was a significant improvement (*P* < .01) in both Group A and Group B after 12 weeks regarding the change in stool properties using the Bristol score.

The change in stool properties was observed using the Bristol score. Before the intervention, Group A and Group B had similar scores of 1.3 and 1.4, respectively. However, after 12 weeks, there was a significant improvement (*P* < .01, paired *t* test) in both groups with scores of 4.4 in Group A and 3.7 in Group B (Fig. 4), and the amount of defecation increased. In addition, because few patients had a history of anorexia in this survey, improvement in abdominal bloating was investigated, which concluded that it was extremely effective in improving symptoms.

**Figure 5:**
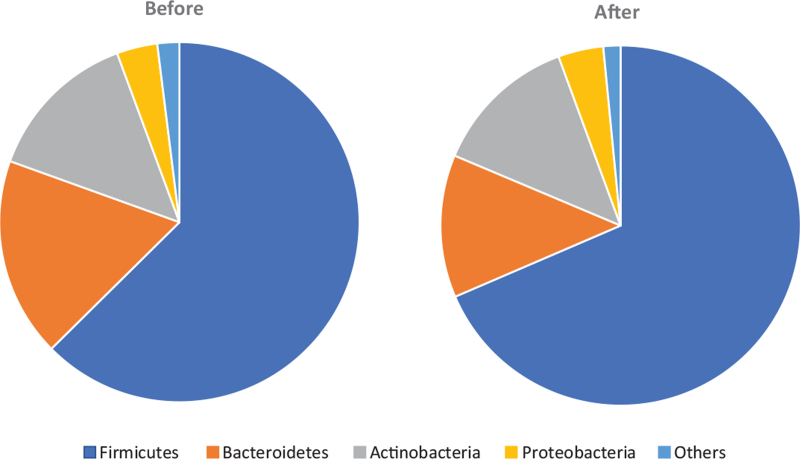
There was a decrease in the Bacteroidetes phyla and a tendency for the Firmicutes phylum to increase by a similar amount, although no significant alternation was observed in the intestinal bacteria at the phylum level.

Lastly, we examined whether changes could be observed in the intestinal flora as constipation improved in Group A. After examining any alternation in the intestinal bacteria at the phylum level, no significant change in the number of strains and composition of Actinobacteria and Proteobacteria was found. However, the number of Bacteroidetes phyla decreased slightly, and the Firmicutes phylum also increased by a similar amount (Fig. 5).

## 4 Discussion

Whether intervention was conducted using only oral constipation-improving drugs or laxatives combined with probiotics, it was extremely effective in improving spontaneous defecation, the Bristol score, and symptoms of patients. There was no clear difference in the final validation results after 12 weeks between Groups A and B; however, the probiotic combination group (B) experienced early improvements in constipation. When examined at the phylum level, no significant difference was found in the results of intestinal flora, which included primarily 4 types: Firmicutes, Proteo-bacteria, Actino-bacteria, and Bacteroidetes phyla.^[11]^ However, in patients in the intervention group (Group A) who were administered the constipation drug only, there was a slight decrease in the Bacteroidetes phyla and a tendency for the Firmicutes phylum to increase by the same amount. These results indicate that improving constipation might lead to a favorable change in the bacteria phylum since the Bacteroidetes phyla may cause opportunistic infections.

Through an evaluation of interventions for treating elderly patients complaining of constipation, significant improvement in early spontaneous defecation was recognized when probiotics were added to the laxative, suggesting that it may be a more effective treatment. In terms of the age-related changes in salvaging intestinal flora in human studies, it has been reported that there is a certain tendency for Proteo-bacteria to increase and Firmicutes to decrease at the genus level. The intestinal tract does not only regulates digestion and absorption to control systemic nutrition but also constitutes the largest immune system in humans.^[12]^ It has been confirmed that intestinal bacteria and intestinal immunity are closely related^[13]^ and significantly influence systemic immunity.^[14]^ In other words, existing literature suggests that the increase of harmful bacteria in the intestine in the elderly could be related to the decrease of systemic immunity and its susceptibility to infection. The results of our study suggest that relieving constipation may cause changes in the bacterial phylum and could contribute to an improvement in the intestinal environment since the Bacteroidetes phyla that may cause opportunistic infections tended to decrease. In the future, we intend to examine changes in the intestinal flora using a constipation drug with probiotics stored in our biobank to verify whether further improvement is observed. Recent studies have gradually revealed the relationship between substances produced by harmful intestinal bacteria and lifestyle-related diseases, such as hypertension, diabetes, and dementia.^[15–17]^ Considering our research results in conjunction with the conclusions of the studies mentioned above, it may be concluded that facilitating a more favorable intestinal environment by relieving constipation is expected to help prevent the onset of various characteristic diseases of the elderly in addition to diseases, such as pneumonia due to immunosuppression. Only a small number of patients who had constipation were verified and participated in this study. Further research should be extended to a larger sample to establish whether changes in intestinal flora are related to the elimination of constipation. Moreover, it is also important to explore the possibility that the combination of laxatives and probiotics may be a more effective treatment for constipation. Furthermore, an ideal intestinal environment with less harmful bacteria induced by treatment of constipation would lead to better intestinal and systemic immunity. Therefore, we plan to conduct a longitudinal study investigating whether maintaining the intestinal flora environment contributes to improving systemic immunity and the future health prognosis of patients.^[18,19]^

## Acknowledgments

We would like to thank the BioBank and NCGG for quality control of the clinical samples.

We would also like to thank Editage (www.editage.com) for English language editing.

## Author contributions

**Conceptualization:** Toshihiro Matsuura.

**Data curation:** Toshihiro Matsuura.

**Funding acquisition:** Toshihiro Matsuura.

**Investigation:** Toshihiro Matsuura, Kazuhiro Kyokane, Satoru Yamada, Yuji Kuno.

**Methodology:** Toshihiro Matsuura, Kazuhiro Kyokane.

**Project administration:** Toshihiro Matsuura.

**Supervision:** Toshihiro Matsuura.

**Validation:** Toshihiro Matsuura.

**Visualization:** Toshihiro Matsuura.

**Writing – original draft:** Toshihiro Matsuura.

**Writing – review & editing:** Toshihiro Matsuura.
